# 肺部结节及肺癌患者新型冠状病毒感染后肺部手术时机的初步建议

**DOI:** 10.3779/j.issn.1009-3419.2023.102.04

**Published:** 2023-02-20

**Authors:** Xin LI, Ming DONG, Song XU, Honglin ZHAO, Sen WEI, Zuoqing SONG, Minghui LIU, Dian REN, Fan REN, Qingchun ZHAO, Renwang LIU, Chunqiu XIA, Gang CHEN, Jun CHEN

**Affiliations:** 300052 天津，天津医科大学总医院肺部肿瘤外科; Department of Lung Cancer Surgery, Tianjin Medical University General Hospital, Tianjin 300052, China

**Keywords:** 新型冠状病毒感染, 肺肿瘤, 肺部手术, Novel coronavirus infection, Lung neoplasms, Lung surgery

## Abstract

近年来，新型冠状病毒感染疫情给全球医疗、政治、经济领域带来了巨大的影响。自新型冠状病毒感染疫情流行开始以来，我们对新型冠状病毒感染影响的了解有了几何数级的增长，近期国内新型冠状病毒感染疫情变化迅速，对于肺部发现肿瘤性病变患者的外科手术如何开展也因近期疫情的变化而出现了争议。一些研究表明，接受肺癌手术的患者感染新型冠状病毒后较普通患者更易发生呼吸衰竭和围术期死亡事件，然而，癌症治疗的推迟也与患者死亡率的上升有关。特别是新型冠状病毒奥密克戎变异株具有更高的传播性，并有可能逃避通过以前的新型冠状病毒感染、疫苗接种获得的免疫力，为了将手术患者发生新型冠状病毒感染的风险降至最低，有必要制定新的治疗指南、专家共识和预防措施，但目前疫情的快速变化导致我们没有充足的时间和证据来制定指南和共识。胸外科医生急需在术前评估发生严重并发症风险较高的特定患者人群的标准，权衡外科治疗的获益与新型冠状病毒感染的风险孰轻孰重。因此，我们尝试结合在新型冠状病毒感染流行情况下不同地区肿瘤学及胸外科学组织对于肺癌手术的指南及共识，给出一些目前国内疫情期间肺部结节及肺癌患者新型冠状病毒感染后肺部手术时机的初步建议，并希望能引发更深入的讨论和建议。

## Abstract (en)

In recent years, the corona virus disease 2019 (COVID-19) pandemic has had a huge impact on the global medical, political and economic fields. Since the beginning of the COVID-19 epidemic, our understanding of the impact of COVID-19 has grown exponentially. Recently, the COVID-19 epidemic has changed rapidly in China, and there has been controversy over how to carry out surgical operations for patients with lung neoplastic lesions. Some studies have shown that lung cancer patients undergoing surgery are more likely to experience respiratory failure and perioperative death after contracting COVID-19 than the general population, however, delays in cancer treatment are also associated with increased mortality among these patients. In particular, the novel coronavirus Omikron variant has a higher transmissibility and may escape the immunity obtained through the previous novel coronavirus infection and vaccination. In order to minimize the risk of novel coronavirus infection in surgical patients, it is necessary to develop new treatment guidelines, expert consensus and preventive measures. However, the current rapid change of the epidemic situation has led to insufficient time and evidence to develop guidelines and consensus. Therefore, thoracic surgeons need to evaluate specific patient populations at higher risk of severe complications before surgery and weigh the benefit of surgical treatment against the risk of novel coronavirus infection. We try to give some recommendations on lung surgery during the current domestic epidemic situation based on the guidelines and consensus of oncology and thoracic surgery organizations in different regions on lung surgery.

2019年新型冠状病毒感染大流行及其对卫生系统造成的压力极大地改变了全球目前的医学实践。我们已经看到，新型冠状病毒感染患者的病程在表现形式上是多样化的。根据目前的情况来看，新型冠状病毒奥密克戎变异株导致的疾病严重程度与我们最初的认知有所区别，除了可能停留在口咽部和鼻咽部造成轻微症状，也会对有基础疾病、合并症或老年患者的肺部造成浸润和损害，出现非常严重的症状，包括急性呼吸窘迫综合征（acute respiratory distress syndrome, ARDS）和多器官功能障碍，尤其是对于高龄老年患者，出现严重症状导致死亡的情况屡有发生。许多千万级以上人口的大城市成为新型冠状病毒感染流行的中心，导致医疗设备、药品、重症监护室（intensive care unit, ICU）床位和医务人员的短缺。一些初期研究数据^[1,2]^显示，接受肺切除术的非小细胞肺癌（non-small cell lung cancer, NSCLC）患者，围术期感染新型冠状病毒与更高的呼吸衰竭发生率和死亡率相关。但是，对于NSCLC患者延迟手术可能产生的不利影响，目前尚无共识。我们也还不清楚接受手术治疗的肺癌患者是否有必要采取风险缓解措施，如果是的话，这些措施应该包括哪些内容。

事实上，我们应该清楚地认识到，对于肺癌患者而言，新型冠状病毒感染导致的死亡风险在所有癌症患者中最高^[3]^，肺癌已被证明是新型冠状病毒感染发病率和死亡率的独立危险因素^[4]^，但其具体原因尚不十分明确。疫情初期，由于个人防护装备和ICU床位不足，患者数量过多，许多医院被迫推迟手术。当时，关于非新型冠状病毒感染病例管理的数据有限，也没有提出明确的指导方针。对于NSCLC的手术治疗，我们曾提出建议，在个案基础上进行多学科讨论共同商议，以便最终决策^[5]^。

美国外科医师协会（American College of Surgeons, ACS）新型冠状病毒感染诊疗指南将外科治疗患者分为三个分诊级别：半紧急情况（阶段1）：需手术患者中新型冠状病毒感染患者较少，医院资源充足；紧急情况（阶段2），需手术患者中有大量确诊新型冠状病毒感染患者，但ICU容量或医院资源有限；高度紧急情况（阶段3）：几乎所有院内医疗资源都被分配给新型冠状病毒感染患者或医疗资源极度短缺。阶段1，ACS建议推迟主要成分为磨玻璃结节（实性成分<50%）、实性结节直径（最大径）<2 cm或组织学考虑为惰性的NSCLC患者。而直径（最大径）在2 cm以上的实性结节、临床诊断伴有淋巴结转移的NSCLC患者以及新辅助治疗后的患者都需要尽快治疗。阶段2，指南建议推迟所有择期胸外科手术。对于考虑出现肿瘤相关感染及手术并发症的患者，若血流动力学相对稳定，则应予以治疗。阶段3，ACS建议推迟所有手术，除了临床判断患者出现气道梗阻、肿瘤相关败血症或血流动力学不稳定的手术并发症患者^[6]^。

欧洲肿瘤内科学会（European Society for Medical Oncology, ESMO）肺癌手术指南将确诊新型冠状病毒感染的需手术的胸外科患者分为三个优先级：高、中、低。高优先级的手术包括胸腔积液引流合并或不合并胸膜固定术、心包积液开窗引流术等。此外，新辅助化疗后的T_2_N_0_患者、可切除的T_3_/T_4_原发肿瘤和可切除的N_1_/N_2_患者也都被认为是高优先级。中优先级手术包括可切除的T_1a_N_0_患者或考虑可能为恶性病变的需行活检患者。此外，具有以下特征的结节的诊断检查或手术治疗也被认为是中等优先级：实性结节或亚实性结节中的实性成分>500 mm^3^、胸膜实性结节直径（最大径）>1 cm、结节体积倍增时间<400天或磨玻璃结节中出现新发实性成分。低优先级手术包括可能的良性病变、纯磨玻璃结节的活检以及体积>500 mm^3^的结节的诊断性切除或结节体积倍增时间>600天^[7]^。

参考已经发表的部分诊疗指南，结合国内目前新型冠状病毒感染病例大幅增长的现状，我们对需要手术的临床诊断为肺癌的患者给出如下建议：

建议1：经常规检查[包括但不限于正电子发射计算机断层显像（positron emission tomography-computed tomography, PET-CT）或经皮肺穿刺活检]提示为良性病变或可能为恶性病变但原发病灶直径（最大径）<2 cm、原本计划行择期手术治疗的患者，建议患者暂停手术，随诊观察（不短于3个月），待疫情流行期结束或相对稳定后再行择期手术治疗。

建议2：经常规检查（包括但不限于PET-CT或经皮肺穿刺活检）提示为恶性病变，且原发病灶直径（最大径）>2 cm且<3 cm，伴淋巴结转移患者建议先接受新辅助治疗；不伴淋巴结转移患者，建议接受新辅助治疗或原发病灶立体定向放射治疗（stereotactic body radiotherapy, SBRT），以上建议适合治疗前取得明确病理的患者。若治疗前无法取得明确病理，而影像学提示伴随同侧肺门、纵隔淋巴结转移患者可经多学科诊疗（multi-disciplinary treatment, MDT）决策后予手术治疗，不伴随淋巴结转移患者建议可临床观察，延缓手术。

建议3：经常规检查（包括但不限于PET-CT或经皮肺穿刺活检）提示为恶性病变，且原发病灶直径（最大径）≥3 cm，也认为属于高优先级手术，经MDT决策后可予以手术治疗或根据具体情况予以新辅助治疗。

建议4：高优先级手术，如中心型病变伴随大量咯血或病变累及主气道伴随严重气道梗阻的患者，经MDT决策后，应予以手术治疗。

建议5：对于纯磨玻璃结节或亚实性结节（实性成分<50%）为主的病灶，建议随诊观察（不短于3个月），若结节在观察期内出现直径增大>20%，经MDT后考虑病变活跃的患者，可予手术治疗，但需向患者及家属交代，手术建议是基于疾病现状而非指南建议。

建议6：目前旨在减少患者在围术期感染新型冠状病毒风险的防护措施应该继续执行，而且鉴于奥密克戎变异株的传播性增加，应该加强这些措施（如呼吸道防护设备及单间病房治疗）。

建议7：仍建议患者在术前进行核酸检测，如果患者在计划手术日期的7周内检测出新型冠状病毒感染阳性，患者应通知手术医生。从此时起，手术团队和患者之间应根据病情就推迟手术的风险和获益进行评估和对话。一般建议阳性患者核酸转阴6周-8周再考虑行手术治疗。

建议8：如果经MDT决策认为有必要在风险可能增加的时期进行手术，那么这些潜在的风险应该包括在知情同意书中，并与患者及家属共同决策。

尽管我们对新型冠状病毒的了解越来越多，相关的研究也越来越多，但许多问题仍然没有答案。对于肺部结节或肺癌的外科治疗，手术延迟的长期流行病学影响还有待观察，国内已有胸外科专家联合提出疫情期间胸外科手术风险管控的几点建议^[8]^，我们倡导更多的胸外科同道根据国内疫情和防控政策的变化进行更为广泛和深入的讨论，并将关于胸外科手术的建议进行随时更新。

目前医疗资源向新型冠状病毒感染患者的大量倾斜，是否可能会对非新型冠状病毒相关的严重疾病（比如恶性肿瘤）造成过度延误，从而对患者的生存产生负面影响，也是我们亟待解决的问题。全球均需要更大样本量的数据来为我们提供更多关于在新型冠状病毒感染大流行期间如何量身定制肺部结节或肺癌治疗的见解。我们目前的共识是新型冠状病毒感染会造成肺部结节或肺癌患者围术期并发症和死亡率增加，但其病理生理机制仍需要进一步研究。
